# Infant gut microbiota modulation by human milk disaccharides in humanized microbiome mice

**DOI:** 10.1080/19490976.2021.1914377

**Published:** 2021-05-03

**Authors:** Antonio Rubio-Del-Campo, Roberto Gozalbo-Rovira, Eva M. Moya-Gonzálvez, Juan Alberola, Jesús Rodríguez-Díaz, María J. Yebra

**Affiliations:** a Laboratorio de Bacterias Lácticas y Probióticos, Departamento de Biotecnología de Alimentos, IATA-CSIC, Paterna, Spain; b Departamento de Microbiología, Facultad de Medicina, Universidad de Valencia, Valencia, Spain

**Keywords:** fucosyl-α-1, 3-*N*-acetylglucosamine, fucosyl-α-1, 6-*N*-acetylglucosamine, lacto-*N*-biose, galacto-*N*–biose, human milk oligosaccharides, infant fecal microbiota, humanized mouse model, short-chain fatty acids, cytokines

## Abstract

Human milk glycans present a unique diversity of structures that suggest different mechanisms by which they may affect the infant microbiome development. A humanized mouse model generated by infant fecal transplantation was utilized here to evaluate the impact of fucosyl-α1,3-GlcNAc (3FN), fucosyl-α1,6-GlcNAc, lacto-*N*-biose (LNB) and galacto-*N*-biose on the fecal microbiota and host–microbiota interactions. 16S rRNA amplicon sequencing showed that certain bacterial genera significantly increased (*Ruminococcus* and *Oscillospira*) or decreased (*Eubacterium* and *Clostridium*) in all disaccharide-supplemented groups. Interestingly, cluster analysis differentiates the consumption of fucosyl-oligosaccharides from galactosyl-oligosaccharides, highlighting the disappearance of *Akkermansia* genus in both fucosyl-oligosaccharides. An increment of the relative abundance of *Coprococcus* genus was only observed with 3FN. As well, LNB significantly increased the relative abundance of *Bifidobacterium*, whereas the absolute levels of this genus, as measured by quantitative real-time PCR, did not significantly increase. OTUs corresponding to the species *Bifidobacterium longum, Bifidobacterium adolescentis* and *Ruminococcus gnavus* were not present in the control after the 3-week intervention, but were shared among the donor and specific disaccharide groups, indicating that their survival is dependent on disaccharide supplementation. The 3FN-feeding group showed increased levels of butyrate and acetate in the colon, and decreased levels of serum HDL-cholesterol. 3FN also down-regulated the pro-inflammatory cytokine TNF-α and up-regulated the anti-inflammatory cytokines IL-10 and IL-13, and the Toll-like receptor 2 in the large intestine tissue. The present study revealed that the four disaccharides show efficacy in producing beneficial compositional shifts of the gut microbiota and in addition, the 3FN demonstrated physiological and immunomodulatory roles.

## Introduction

Microbial colonization of the infant gastrointestinal tract plays a fundamental role in maintaining a healthy status.^1,2^ Disruption or inappropriate development of the neonatal gut microbial community is linked to diseases such as necrotizing enterocolitis, diarrhea and allergies.^3–5^ The postnatal microbiota structure of the infant gut is closely related to the mother’s breast milk microbiota and the unique human milk glycan composition.^6–8^ This includes free oligosaccharides (HMOs), mucins, glycoproteins and glycolipids.^9,10^ HMOs constitute the third largest solid component of human milk and over 200 different structures have already been identified.^9^ Fecal microbiota of breastfed infants is generally dominated by the phylum Actinobacteria, with *Bifidobacterium* as the main genus, and Firmicutes, with diverse representation from numerous genera.^11^ Several studies have demonstrated that the fermentation of HMOs and the glycan moiety of mucins stimulate the growth of specific strains belonging to the genus *Bifidobacterium* and to a lesser extent to *Lactobacillus*.^10^ HMOs are also associated with various beneficial effects, including immunomodulation, protection against infectious diseases, stimulation of intestine barrier functions and brain development.^12–14^ Compared to human milk, infant formula contains low amounts of oligosaccharides with limited structural diversity and fucosylation.^15^ Indeed, the gut microbiome composition and health outcomes of formula-fed infants differ markedly from that of exclusively breastfed infants.^16^ Therefore, it will be important to identify glycan structures that are crucial for developing a beneficial gut microbiota composition, and that can be easily produced for using individually or combined in a glycan mix.

A few studies using *in vivo* animal models highlight the ability of individual HMOs to modify the composition of the gastrointestinal microbiota. The levels of *Barnesiella* in the gut increased in a baby mouse model exposured to 2ʹ-fucosyllactose (2ʹFL) and 3-fucosyllactose (3FL), and these changes in the microbiota affected the susceptibility of mice to dextran sulfate sodium-induced colitis.^17^ Supplementation of 2ʹFL also changed the composition of cecal microbiota, improved metabolic profiles and gut-brain signaling, and downregulated the expression of pro-inflamatory cytokines in a high-fat fed mouse model.^18^ Mice fed with a diet containing 3ʹ-sialyllactose (3’SL) and 6ʹ-sialyllactose (6’SL) have lesser colonic microbiota alterations and better anxiety-like behavior than control mice fed with a standard diet.^19^ Humanized microbiome animal models are emerging as powerful tools for analyzing human microbiota in a controlled mode. The microbial populations of fecal and jejunal content of germ-free mice inoculated with a model of human baby microbiota, which comprises seven bacterial strains, were modulated with galactosyl-oligosaccharides co-administrated with the probiotics *Lactobacillus paracasei* or *Lactobacillus rhamnosus*.^20^ An increase in the number of specific *Bifidobacterium* species and a reduction of *Clostridium perfringens* was observed. In addition, host metabolic pathways as lipid profiles and gluconeogenesis among others were modulated.^20^ An important reduction of clostridia numbers was observed recently in a germ-free mouse model with fecal microbiota from infants born by cesarean section and in the presence of a combination of *Bifidobacterium infantis* and human milk or HMOs.^21^

**Figure 1.**
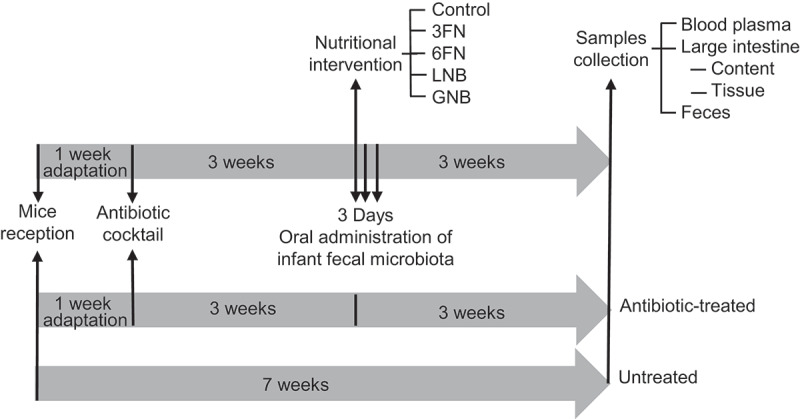
Schematic representation of the experimental design. *n* = 5 mice in each group: untreated, antibiotic-treated, control, 3FN (fucosyl-α1,3-GlcNAc), 6FN (fucosyl-α1,6-GlcNAc), LNB (lacto-*N*-biose) and GNB (galacto-*N*-biose)

The disaccharides fucosyl-α1,3-GlcNAc (3FN) and fucosyl-α1,6-GlcNAc (6FN) that form part of HMOs and core-fucosylated *N*-glycans, respectively, have been synthetized in our laboratory.^22,23^ *In vitro* fermentation analysis, using pure cultures^23^ and batch cultures with infant fecal microbiota,^24^ has demonstrated that 3FN stimulates the growth of the species *Lactobacillus casei* and *Bifidobacterium breve*, respectively. We have also produced lacto-*N*-biose (LNB; Gal- β1,3-GlcNAc), the main building block of type-1 HMOs, and galacto-*N*-biose (GNB; Gal- β1,3-GalNAc), the core type-1 sugar from mucins.^25^ LNB is also present as free sugar in human milk.^26^ Both disaccharides have been shown to efficiently promote *in vitro* the growth of specific *Bifidobacterium* species.^24,27^ In this work, a humanized mouse model, which was generated by infant fecal microbiota transplantation, was utilized to evaluate the effects of 3FN, 6FN, LNB and GNB on the development of the gut microbiota composition (Figure 1). Short-chain fatty acids production and interactions host–microbiota, through the assessment of serum lipid profile and cytokines expression, were also measured.

## Results

### Modification of the fecal murine microbiota by infant fecal microbiota transplantation

**Figure 2.**
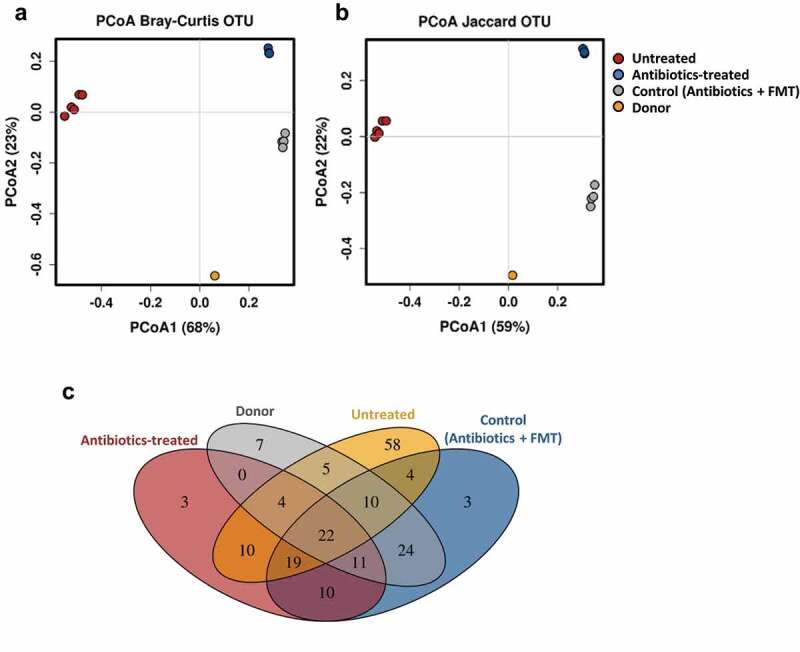
Fecal microbial diversity of mice in response to antibiotic-treatment and infant fecal transplantation. Principal coordinates analysis (PCoA) plot of fecal microbiota composition using Bray–Curtis (a) and Jaccard (b) distance metrics. (c) Venn diagram of shared OTUs between the infant donor fecal mix and the fecal microbiota of the untreated, antibiotic-treated and control mice groups. FMT, fecal microbiota transplantation

We first assessed if the antibiotic treatment followed by infant fecal microbiota transplantation shifts the murine microbiota composition toward the donor microbiota profile. A PCoA plot of the 16S rRNA fecal microbial composition data using Bray–Curtis and Jaccard distance metrics showed that the mice groups, untreated, treated with antibiotics and treated with antibiotics followed by oral administration of infant fecal microbiota (control), and the pooled donor feces clustered separately (Figure 2a,b). Both mice groups, treated with antibiotics and control, were separated from the untreated mice group along the PCoA1 axis and from each other along the PCoA2 axis. The significant separation between the antibiotics-treated and control mice groups was confirmed by statistical analysis (ANOSIM *R* = 1, *p* = 0.025; Adonis *R*^2^ = 0.919, *p* = 0.029). The control mice group has a lower distance to the donor microbiota than the mice group only treated with antibiotics (Figure 2a, b). A Venn diagram showed that 24 OTUs were absent from untreated and antibiotics-treated mice groups and shared by the pooled infant donor and the control humanized mice group (Figure 2c and Supplemental Table 1). They belonged to the genera *Bacteroides, Parabacteroides, Phascolarctobacterium, Klebsiella* and *Eggerthella*, and the families *Ruminococcaceae* and *Enterobacteriaceae*. In addition, ten OTUs from the genera *Bacteroides, Parabacteroides* and *Phascolarctobacterium* were present in all the groups except in the antibiotic-treated group, indicating that their presence in the control group, which is also treated with antibiotics, is due to the transplanted infant microbiota and not due to the indigenous bacteria. All these results revealed that antibiotic treatment followed by oral inoculation led to successful engraftment of the infant microbiota. However, a limitation of this humanized animal model is that seven OTUs (belonging to the genera *Bifidobacterium, Collinsella, Lactobacillus* and *Streptococcus*, and to the order Clostridiales) specific to the donor were not transferred to the recipient mice. As well 19 OTUs (belonging to the genera *Lactococcus* and *SMB53*, the families *Clostridiaceae, Peptostreptococcaceae* and *Rikenellaceae*, and to the order Clostridiales) were shared by the untreated, antibiotic-treated group and the control humanized group, indicating that a proportion of the indigenous bacteria remain in the transplanted mice (Figure 2c and Supplemental Table 1). It cannot be ruled out that some of the residual microbiota influence the effects of the disaccharides, described below, on the infant gut microbiota.

### Human milk-associated disaccharides produce compositional changes in the gut microbiota

**Figure 3.**
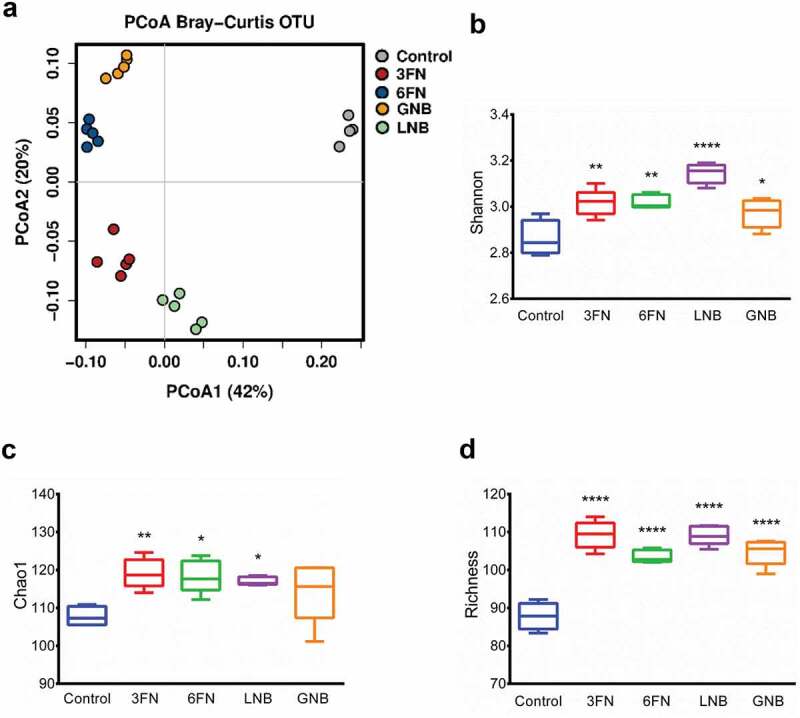
Fecal microbial diversity and richness of infant fecal transplanted mice in response to disaccharide-supplemented diets. Principal coordinates analysis (PCoA) plot of fecal microbiota composition using Bray–Curtis (a), Shannon index (b), Chao1 index (c) and absolute richness (d) at OTU level. Box plots present the median (interquartile range) and min/max. 3FN (fucosyl-α1,3-GlcNAc), 6FN (fucosyl-α1,6-GlcNAc), LNB (lacto-*N*-biose) and GNB (galacto-*N*-biose). Statistical significant differences compared to control are indicated: *p < 0.05; **p < 0.01; ***p < 0.001, ****p < 0.0001

The effect of the consumption of the disaccharides 3FN, 6FN, LNB and GNB was tested in mice pre-treated with antibiotics followed by infant fecal transplantation. To understand the global changes of fecal microbial composition due to the consumption of these disaccharides, PCoA analysis was performed using Bray–Curtis distance metrics. The results showed that the control and each disaccharide feeding groups clustered separately (Figure 3a). The consumption of the disaccharides has a strong effect on the fecal microbiota as PCoA 1 and PCoA 2 axes explain 42% and 20% of the total variance, respectively, and it was confirmed by statistical analysis (ANOSIM *R* = 0.992, *p* = 0.001; Adonis *R*^2^ = 0.829, *p* = 0.0003). The microbiota α-diversity of all disaccharide-supplemented mice groups was significantly higher than that in control mice, with the only exception of the Chao1 index for the GNB group (Figure 3b, c, d). Therefore, these results indicate that each of the disaccharides evaluated here is able to modify the fecal bacterial community diversity and richness.

**Figure 4.**
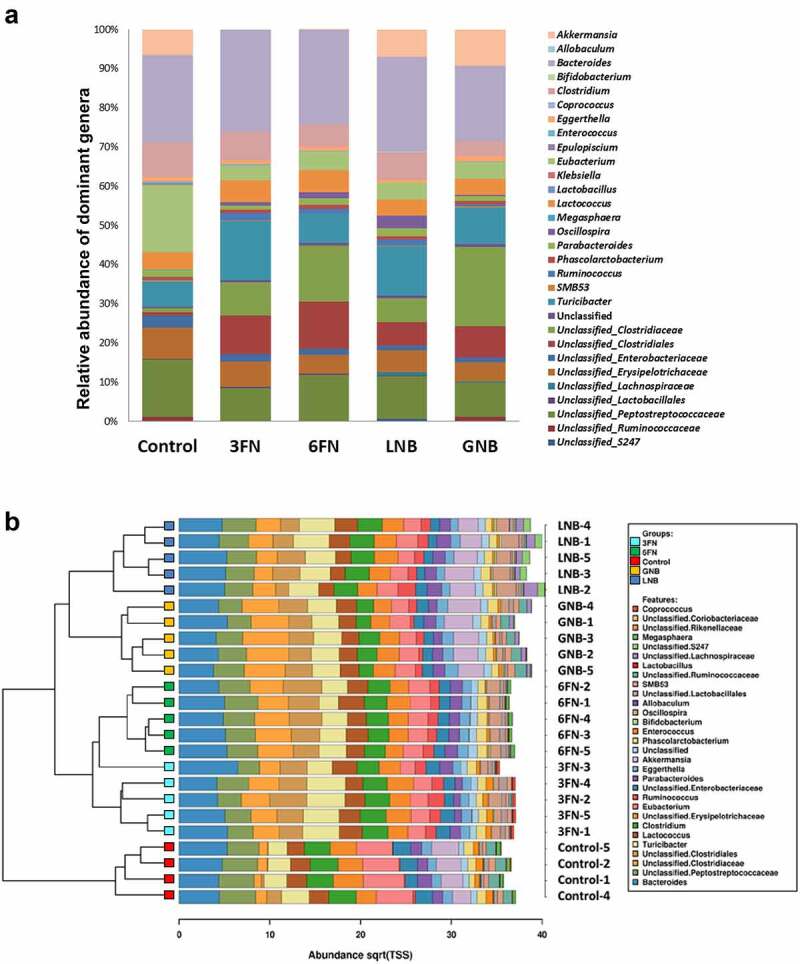
Fecal microbial composition of infant fecal transplanted mice in response to disaccharide-supplemented diets. (a) Fecal microbial relative abundances at genus level. Bars represent each diet group and values are mean relative abundance of each bacterial genus. (b) Clustered bar-chart analysis of fecal mice samples at genus level. Bars represent each mouse. Control group (*n* = 4); diet groups (*n* = 5). 3FN (fucosyl-α1,3-GlcNAc), 6FN (fucosyl-α1,6-GlcNAc), LNB (lacto-*N*-biose) and GNB (galacto-*N*-biose)

**Figure 5.**
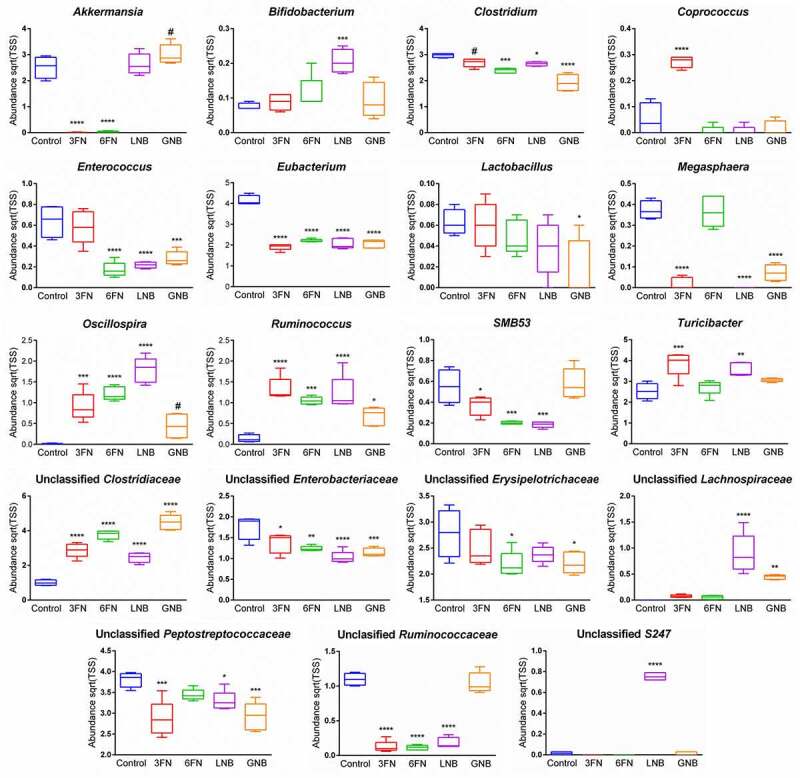
Abundances of the fecal bacterial genera that showed statistically significant differences for at least one of the disaccharide-supplemented mice groups compared to control group. 3FN (fucosyl-α1,3-GlcNAc), 6FN (fucosyl-α1,6-GlcNAc), LNB (lacto-*N*-biose) and GNB (galacto-*N*-biose). Box plots present the median (interquartile range) and min/max. *n* = 4 (control group); *n* = 5 (diet group). Statistical significant differences compared to control are indicated: #*p* < 0.1, *p < 0.05; **p < 0.01; ***p < 0.001, ****p < 0.0001

Analysis of the relative abundance of fecal microbiota at the family level identified 12 families that were differentially abundant between at least one of the four disaccharide-fed mice groups and the control group (Supplemental Figure 1). Notably, LNB supplementation significantly increased the abundance of *Bifidobacteriaceae, Lachnospiraceae, Ruminococcaceae*, S247 and *Turicibacteraceae*. The four disaccharides increased *Clostridiaceae* and unclassified *Clostridiales*, and significantly decreased *Enterobacteriaceae* and *Erysipelotrichaceae* with respect to the control without carbohydrate supplementation. Regarding the relative abundance of fecal microbiota at the genus level, the results showed differences among the five mice groups (Figure 4). Interestingly, a clustered bar-chart analysis with the top 30 more abundant genera showed two main clusters that differentiate control group from disaccharide-treated groups, and within these two main clusters the analysis clearly differentiates the consumption of fucosyl-oligosaccharides from the consumption of galactosyl-oligosaccharides (Figure 4b). Relative abundance changes of 19 genera were significantly associated with at least one of the four disaccharide-supplementation mice groups with respect to the control group (Figure 5). The relative abundance of *Bibidobacterium* and unclassified S247 was significantly increased by LNB and *Lactobacillus* abundance decreased with GNB. *Coprococcus* abundance increased by 3FN, and an unclassified genus of the *Lachnospiraceae* family was significantly increased by both, LNB and GNB. Mice fed with any of the fucosyl-disaccharides tested here showed a significantly decrease in abundance of *Akkermansia* while in GNB-fed mice its abundance increased. 3FN and LNB-supplemented mice groups showed an increase in *Turicibacter* abundance, and 6FN and GNB a decrease in unclassified *Erysipelotrichae*. The abundance of *Megasphaera, Enterococcus, SMB53*, unclassified *Peptostreptococcaceae* and unclassified *Ruminococcaceae* decreased in three out of the four disaccharide-fed mice groups. Certain bacterial genera were affected by all four treatments. Thus, the relative abundance of *Ruminococcus, Oscillospira* and an unclassified genus of the *Clostridiaceae* family was significantly increased with the four disaccharides tested (Figure 5). Contrarily, the genus *Eubacterium*, an unclassified genus of the *Enterobacteriaceae* family and *Clostridium* decreased with the four disaccharides.

**Figure 6.**
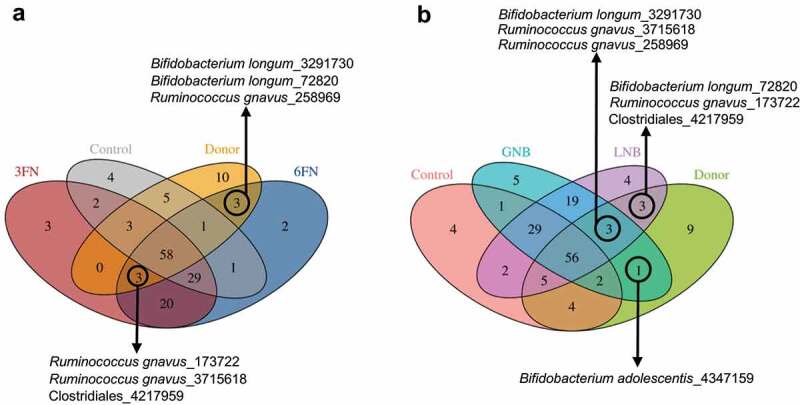
Venn diagram of shared OTUs between the infant donor fecal mix, the fecal microbiota of the control and fucosyl-oligosaccharides (a) or galactosyl-oligosaccharides (b) mice groups. 3FN (fucosyl-α1,3-GlcNAc), 6FN (fucosyl-α1,6-GlcNAc), LNB (lacto-*N*-biose) and GNB (galacto-*N*-biose)

In order to evaluate if the disaccharides promote selectively the growth of bacteria from the donor, we compared the fecal microbiota data in a Venn diagram (Figure 6 and Supplemental Table 2). The results showed that six OTUs corresponding to the species *Bifidobacterium longum* and *Ruminococcus gnavus*, and to the order Clostridiales are shared among the donor and specific disaccharide groups, indicating that their survival possibly relies upon disaccharide supplementation. As well, one OTU belonging to *Bifidobacterium adolescentis* species is only shared by the donor and the GNB group, and it is not present in the control group (Figure 6b). This result suggests that the persistence of this species depends on the GNB supplementation.

### Effects of human milk-associated disaccharides on short-chain fatty acid (SCFA) concentrations in the colon

**Figure 7.**
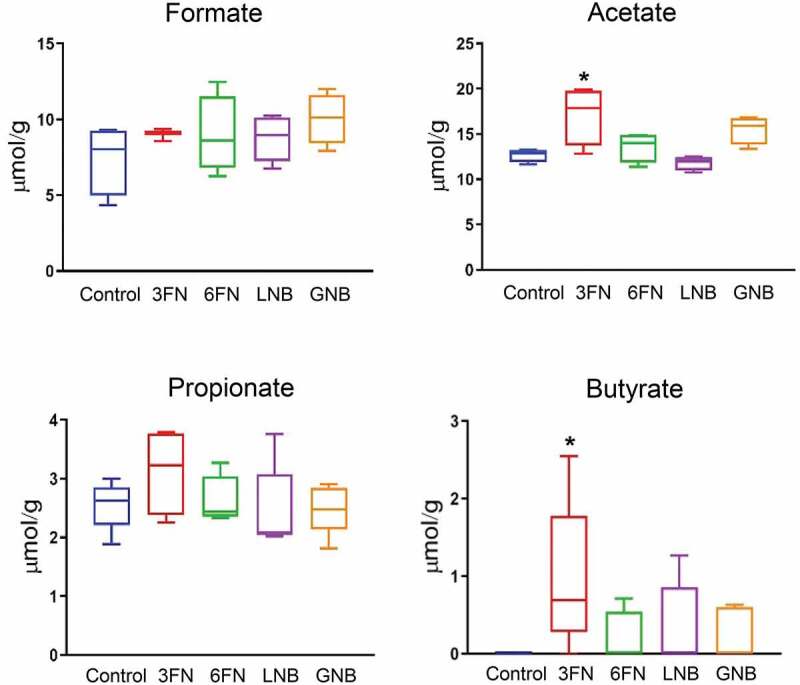
Effect of 3FN (fucosyl-α1,3-GlcNAc), 6FN (fucosyl-α1,6-GlcNAc), LNB (lacto-*N*-biose) and GNB (galacto-*N*-biose) on short-chain fatty acid concentrations in large intestine content of infant fecal transplanted mice. Box plots present the median (interquartile range) and min/max. *n* = 5 (control group); *n* = 5 (diet groups). Statistical significant differences compared to control are indicated: *p < 0.05

SCFAs are the major end products of the carbohydrate fermentation processes by the intestinal microbiota. Acetate, propionate and butyrate represent more than 90% of the total SCFA produced in the colon.^28^ To determine whether supplementation of the specific human milk disaccharides tested here affected microbial metabolic activity, those colonic SCFAs were measured (Figure 7). Formate, a main fermentation end-product,^29^ was also analyzed. None of the assayed disaccharides affected significantly the production of formate and propionate by the microbiota. However, acetate levels increased significantly in the 3FN supplemented mice group compared to the control group. Interestingly, butyrate was not detected in the fecal samples of the control group; however, consumption of the disaccharides evaluated here resulted in butyrate production by the microbiota, being statistically significant for the 3FN feeding group (Figure 7).

### Impact of human milk-associated disaccharides on lipid metabolism

**Figure 8.**
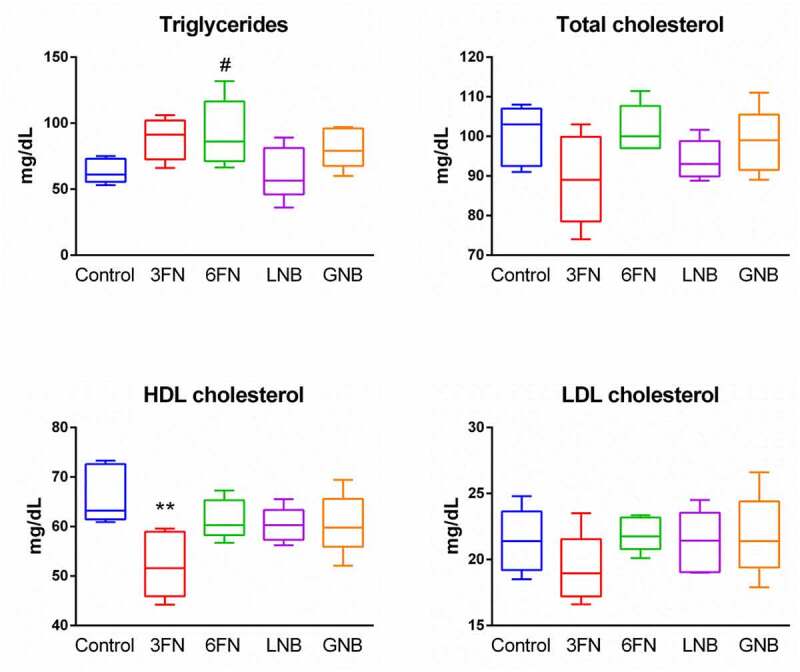
Effect of 3FN (fucosyl-α1,3-GlcNAc), 6FN (fucosyl-α1,6-GlcNAc), LNB (lacto-*N*-biose) and GNB (galacto-*N*-biose) on serum lipid profile of infant fecal transplanted mice. Box plots present the median (interquartile range) and min/max. *n* = 5 (control group); *n* = 5 (diet groups). Statistical significant differences compared to control are indicated: #*p* < 0.1, *p < 0.05; **p < 0.01

Changes in the gut microbial composition and derived metabolites have been shown to influence lipid metabolism.^30,31^ Therefore, we analyzed the effect of the oligosaccharides on body weight, and concentrations of serum triglycerides and cholesterol. At the end of the experimental procedure (week 7), the mice groups supplemented with 3FN and GNB had significantly higher weight gain as percentage of initial body weight in comparison with control mice group (Supplemental Figure 2). The 6FN-feeding group presented significantly higher levels of triglycerides than the control group, and mice consuming 3FN showed significant decreased levels of HDL-cholesterol (Figure 8). None of the tested disaccharides resulted in a significant reduction of total cholesterol and LDL-cholesterol levels. The results obtained here may be relevant since breastfed infants have higher levels of triglycerides, total cholesterol and LDL-cholesterol than formula-fed infants.^32,33^ Unexpectedly, in spite of their high serum lipid concentration, long-term breastfed infants have lower cardiovascular risk in adulthood than their formula-fed counterparts.^34^

### Effects of human milk-associated disaccharides on immunological biomarkers expression at intestinal tissue level

**Figure 9.**
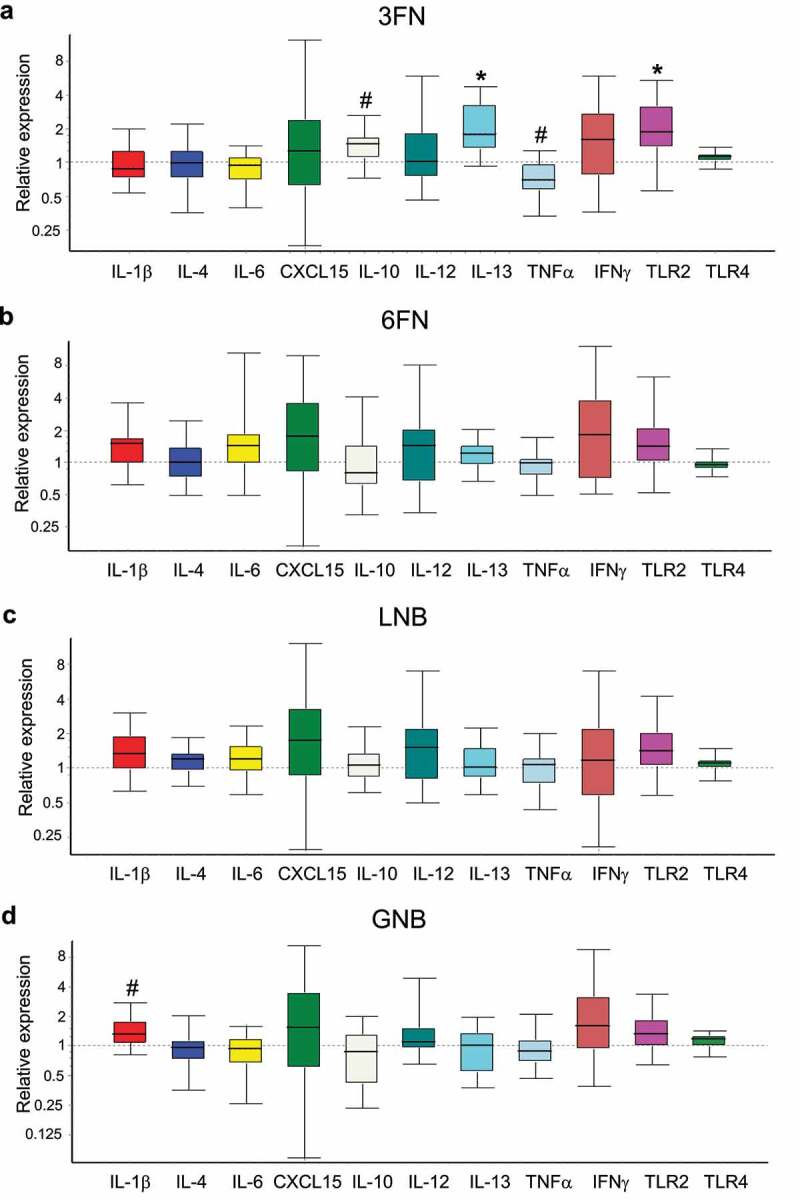
Effect of 3FN (fucosyl-α1,3-GlcNAc) (a), 6FN (fucosyl-α1,6-GlcNAc) (b), LNB (lacto-*N*-biose) (c) and GNB (galacto-*N*-biose) (d) on gene expression of cytokines and Toll-like receptors in the large intestine tissue of infant fecal transplanted mice. The values, expressed as fold-changes, represent relative expression in treated mice groups compared to control group. Box plots present the median (interquartile range) and min/max. *n* = 5 (control group); *n* = 5 (diet groups). Statistical significant differences compared to control are indicated: #*p* < 0.1, *p < 0.05

The expression of genes encoding pro-inflammatory (IL-1β, IL-6, Cxcl15 (IL-8), IL-12, TNF-α, IFN-γ) and anti-inflammatory (IL-4, IL-10 y IL-13) cytokines, and Toll-like receptors (TLR2 y TLR4) were evaluated in the large intestine tissue (Figure 9). Supplementation with 6FN or LNB did not modify significantly the expression of any of those genes involved in the activity of the immune system. However, the supplementation of the disaccharide 3FN resulted in a significant increase in expression of IL-10 and IL-13 and a significant decrease of TNF-α with respect to the control mice group. Moreover, the expression of the gene encoding TLR2, which is involved in the immune response mediated by gut microbiota, is significantly increased in the 3FN mice group (Figure 9a). The other three feeding groups, 6FN, LNB and GNB, showed a trend (*p* = 0.182, 0.164 and 0.171, respectively) toward increased expression levels of that TLR gene (Figure 9b, c, d). The cytokine IL-1β was up-regulated in the GNB supplemented mice group compared to the control group (Figure 9d).

## Discussion

The gut commensal microbiota and associated metabolic products from breastfed infants have long been considered as contributors to infant health.^16^ However, substantial differences have been found between the gut microbiota composition of formula-fed and breastfed infants.^16,35^ Those differences are due in part to the low concentrations and different structures of oligosaccharides found in infant formulas compared to human milk.^15^ Infant formulas are based in bovine milk, which does not contain type-1 oligosaccharides,^15^ lacking therefore the predominant LNB structural unit of HMOs. Another difference with human milk is that oligosaccharides from bovine milk are highly sialylated and to a lesser extent fucosylated. While about 70% of HMOs are fucosylated, only 1% of the oligosaccharides from bovine milk are fucosylated.^36^ Therefore, there is a need to search for functional carbohydrates that can fulfill the oligosaccharide scarcity from infant formulas. In this work, we established a humanized mouse model to evaluate the impact of the disaccharides LNB, GNB and two fucosyldisaccharides, 3FN and 6FN, on the gut microbiota composition and host–microbiota interactions. Germ-free mice colonized with infant fecal microbiota have been previously used to evaluate the effect of probiotics and synbiotic mixes.^20,21,37^ Since germ-free mice have many biological and technological limitations,^38,39^ a strategy using conventional mice treated with antibiotics^40^ followed by oral administration of infant fecal microbiota was successfully used here. There are some host factors that may impact the mice bacterial colonization from infant donors, indeed intestinal mucin glycans that are utilized by some bacterial species as growth substrates differ between humans and mice.^41^ As well, the mucus thickness and many immunological functions are affected by the age of the mice.^42^ In our study, we have used young mice, but whether differences in the mucus layer and the immune system due to mice age would affect the ability of infant gut bacteria to colonize mice merits further research. In the mice model used here, 3 weeks after the infant fecal transplantation about 41% OTUs of the infant donor pool remain in the transplanted mice (Figure 2c). Additionally, seven OTUs were just present in the donor sample and in at least one of the disaccharide-supplemented groups (Figure 6). These results evidenced the suitability of the infant microbiota-associated mice as a model to study the role of human milk glycans on infant gut microbiota development.

Unlike formula-fed infants, the fecal microbiota of breastfed infants is dominated by bifidobacteria.^43^ Using *in vitro* fermentation analysis, our group and others have demonstrated that LNB is metabolized by infant-gut associated bifidobacteria.^24,27^ Here, this disaccharide is tested for the first time using an *in vivo* model and it has been demonstrated that it significantly increased the relative abundance of the genus *Bifidobacterium* in feces. However, the absolute levels of this genus in the LNB mice group were similar to the control group (Supplemental Figure 3), suggesting that the high relative abundance of *Bifidobacterium* in LNB could be due to reductions in the relative abundances of other taxa. Regarding GNB, two OTUs belonging to *B. longum* and *B. adolescentis* species, respectively, that were present in the infant donor sample, persisted in the GNB supplemented group but not in the control group. These results suggest that this disaccharide might have a role in the survival of particular species or strains of bifidobacteria. *B. longum* species persisted also in the LNB and 6FN diet groups. All the *B. longum* strains tested *in vitro* fermented LNB and all of them contain the gene *lpnA* encoding the GNB/LNB phosphorylase specific not only to LNB if not also to GNB.^44^ Indeed, a *B. longum* strain isolated from fermented cultures with infant gut microbiota was able to grow in the presence of GNB.^24^ The positive effect of LNB, GNB or 6FN in the persistence of *B. longum* species in the gastrointestinal tract is a relevant outcome, since this bacterium has been widely associated with prevention and fighting of several intestinal and immune diseases.^45,46^ Regarding *B. adolescentis* species, it has been shown previously that this species is not able to metabolize 3FN and 6FN.^23^ Unlike these, the utilization of LNB and GNB by *B. adolescentis* has not been tested. Then, we analyzed here if the type strain *B. adolescentis* ATCC 15703 utilizes those disaccharides as substrates and the results showed that this strain can be cultured in the presence of both LNB and GNB (data not shown). Survival of *B. adolescentis* in the GNB diet group might be important for serious infant gastrointestinal disorders such as necrotizing enterocolitis, for which a protection effect has been shown by that species.^47^

*R. gnavus* is a human gut symbiont present at early and adult life stages,^48,49^ and various studies have pointed toward a key role of this species in modulating gut inflammatory responses.^50,51^ The four disaccharides tested here significantly increased the relative abundance of the *Ruminococcus* genus in feces and allowed the persistence of three OTUs corresponding to the species *R. gnavus* (Figures 5 and 6). The genomes of strains belonging to this species harbor several genes encoding for glycosidases potentially implicated in the breakdown of mucin-derived glycans (https://www.ncbi.nlm.nih.gov/genome). Four α-L-fucosidases from two *R. gnavus* species have been recently characterized and they catalyzed the release of α-1,2, α-1,3 or α-1,4-linked fucose.^52^ The presence of fucosidases with α-1,3 specificity in this species is in agreement with its survival in the 3FN supplemented group. The activity of α-fucosidases on 3FN and 6FN will produce the constituent monosaccharides fucose and GlcNAc. The action of specific β-galactosidases on LNB and GNB would generate galactose and the corresponding *N*-acetylhexosamines GlcNAc and GalNAc, respectively. *R. gnavus* has been shown to metabolize all those monosaccharides with the exception of GalNAc.^53^

In recent years, the *Akkermansia* genus has received much attention because of its controversial involvement in human health and disease.^5,54^ Low levels of *Akkermansia muciniphila* in the human intestine have been associated with several diseases, including inflammatory bowel disease, atopic dermatitis and type-2 diabetes.^54^ Conversely, a recent study showed that the relative abundance of this genus in a group of infants with allergic diseases is significantly higher than that in the healthy group.^5^ *A. muciniphila* is known as an intestinal mucin-degrading bacterium, and possibly because of this, the members of this genus have been linked to reduced integrity of the intestinal barrier and infiltration of allergens through the intestinal wall.^55^ Alternatively, a role in maintaining intestinal integrity has also been claimed for that bacterium.^56^ A striking difference has been shown between the galactosyl-disaccharides and the fucosyl-disaccharides tested here in relation to the relative abundance of *Akkermansia* genus in feces (Figure 5). While with LNB and GNB (a mucin-derived disaccharide) the levels of these bacteria remained and significantly increased, respectively, with both fucosyl-disaccharides were significantly reduced. This latest result is in agreement with a previous work that showed that the gut of newborn mice fed with regular core-fucosylated milk *N*-glycan had less abundance of members of the *Akkermansia* spp. than those fed with low-core-fucosylated milk *N*-glycan.^57^

A desirable effect of HMOs is also to protect children from pathogenic diarrhea caused by the intestinal viruses rotavirus and norovirus. Interestingly, we have demonstrated in previous studies that those bacterial groups (*Akkermansia* spp. and *Ruminococcus* spp.) had divergent effects in rotavirus and norovirus susceptibility. *Ruminococcus* spp. correlated negatively with both rotavirus and norovirus IgA antibody titters, showing a lower susceptibility to these two virus infections in individuals with higher amounts of *Ruminococcus* spp. (revealing its potential as antiviral bacteria). Contrarily, the IgA antibody titer to rotaviruses positively correlated with the amounts of *Akkermansia* spp., pointing to this bacterial group as a facilitator of rotavirus infections.^58^

SCFAs, the end products of carbohydrate fermentation by the intestinal microbiota, are efficiently absorbed by the gut mucosa and have important effects on host physiology though their involvement in gene expression regulation and action as signaling molecules.^30^ Acetate, propionate and butyrate are the most abundant SCFAs in the colon, and they are substrates for colonocytes and peripheral tissues.^30,59^ In addition, SCFAs decrease the luminal pH helping to inhibit potential pathogens growth and to increase nutrients absorption.^60^ Butyrate has also been associated with protection against colorectal cancer and atherosclerosis, and it showed immune-modulatory activities.^61,62^ Compared with formula-fed infants, exclusively breastfed infants present a higher proportion of acetate with respect to other SCFAs in the gut,^63^ and this may provide protection against intestinal pathogens and allergic disease.^64,65^ Therefore, it is particularly relevant to provide specific carbohydrates that shift the microbiota toward the production of those catabolic products. In this study, the supplementation with 3FN significantly increased the levels of butyrate and acetate. Interestingly, a significant increment of the abundance of *Coprococcus* genus presented in the 3FN-feeding group clearly differentiates this group from the other three disaccharide-feeding groups (Figure 5). The species belonging to that genus have been described to ferment carbohydrates and produce butyrate and acetate as end products.^66,67^ However, other possibilities for the increment of those SCFAs in the 3FN group cannot be discarded, including cross-feeding of intermediary and end metabolites between different gut bacteria.^66^

HMOs are known to affect the mucosal and systemic immunity of newborns, either directly by interacting with the immune cells or indirectly through the microbiota.^68^ Immunomodulatory activity has been previously described for fucosylated HMOs such as 2ʹFL^69^ and lacto-*N*-fucopentaose.^70^ Here, the supplementation of 3FN significantly decreased the expression of the pro-inflammatory cytokine TNF-α and enhanced the expression of the anti-inflammatory cytokines IL-10 and IL-13 in the mice large intestine tissue (Figure 9a). Therefore, these results suggested an anti-inflammatory potential for 3FN. This oligosaccharide enhanced the production of butyrate, which has been previously involved in immune homeostasis, for example, by reducing the expression of pro-inflammatory cytokines, including TNF-α.^71^ We observed that the butyrate levels correlated positively with the expression of the cytokine IL-10 (*r* = 0.6), while it correlated negatively with TNF-α (*r* = −0.7), showing a trend toward statistical significance (*p* = 0.175 and 0.233, respectively). Interestingly, 3FN forms part of the Lewis x antigen and the presence of Lewis x-type oligosaccharides on the human milk glycoprotein mucin 1 have been shown to interact with the dendritic cell-specific intercellular adhesion molecule-3-grabbing non-integrin (DC-SIGN), a specific C-type lectin on dendritic cells that binds fucose.^72^ DC-SIGN is expressed in the entire gastrointestinal tract of neonates and its interaction with fucosylated oligosaccharides has been suggested to be an important mechanism of human milk to shape the infant immune system.^72,73^ Whether butyrate and/or C-type lectins are involved in the immunomodulatory effects of 3FN requires further investigations. The 3FN feeding group also showed a significant increment of the expression of the gene encoding TLR2. The other three feeding groups, 6FN, LNB and GNB, tended to increase the expression of that TLR compared to the control group, although they did not reach statistical significance. TLR2 stimulation has an important role in protecting gut epithelial barrier function.^74^ This immunological receptor recognizes microorganism-associated molecular patterns, such as peptidoglycan, lipoteichoic acid and exopolysaccharide from Gram-positive bacteria.^75^ Indeed, its activation has been demonstrated for *Bidifobacterium* strains isolated from breastfed infant feces.^76^

Even knowing the many benefits of HMOs in infant health, only 2ʹFL and lacto-*N*-neotetraose are currently added to infant formula, possible because their synthesis is still difficult and expensive.^12^ In this study, we demonstrated that four different disaccharides (3FN, 6FN, LNB and GNB) that are present in HMOs and glycoconjugates of human milk and mucosa, may play a role on infant microbiome building. However, this study may have limitations due to the variability usually observed in animal experimentation, and should therefore be corroborated by further research. Within the four disaccharides, it is important to highlight that LNB increased the relative abundance of *Bifidobacterium* genus, whose high levels is the most outstanding differential characteristic of the gut microbiota in breastfed infants. In addition, the efficacy of 3FN in changing the microbiota concomitant with an increase in SCFA levels and an immunomodulation activity was also demonstrated. These results allowed to gain insights in the mechanisms by which human milk glycans are associated with infant health benefits, and the simple structure of those disaccharides, which facilitates their synthesis, make them good candidates for being utilized in infant functional food development.

## Material and methods

### Animals, infant fecal samples and disaccharides

Thirty-five C57BL/6 J female mice, 6 weeks old, were acquired from Charles River Laboratories (Saint Germain Nuelles, France). They were randomly separated into seven groups of five mice each in individually ventilated cages in an environmentally controlled room, following the standard protocols of the animal facilities and the rules of animal wellness.

The study includes four infants, whose parents were volunteers. Our inclusion criteria were that the infants were healthy, receiving no antibiotic or probiotic treatment, between 1 and 3 months old, and exclusively breastfed. Stool samples were collected in anaerobic jars with Oxoid AnaeroGen anaerobic atmosphere generation system sachets (Thermo Scientific), immediately stored at 4 °C and cryopreserved within the next 12 h as previously described with some modifications.^77^ Briefly, cryopreservation media contained 80% BHI (Brain Heart Infusion, Pronadisa) 2X concentrated supplemented with 0.1% of cysteine and 20% skim milk (200 g/l) (Scharlab). Feces were diluted in cryopreservation media 1:2 (vol/vol) and stored in aliquots at −80°C.

The disaccharides 3FN, 6FN, LNB and GNB were synthesized in our laboratory by enzymatic transglycosylation reactions and purified by high-performance liquid chromatography (HPLC) as previously described.^23,25^

### Experimental design, fecal transplant and treatments

The experimental protocol is outlined in Figure 1. The seven groups of mice (untreated, antibiotic-treated, control, 3FN, 6FN, LNB and GNB) were acclimated in the animal facility for 1 week and then an antibiotic cocktail (0.5 g/l vancomycin, 1 g/l neomycin sulfate, 1 g/l metronidazole, 1 g/l ampicillin) was administered in drinking water ad libitum for 3 weeks to all the groups except the untreated group. That antibiotic combination has been previously used.^40^ The antibiotic cocktail was renewed every 3 d and removed 24 h before infant fecal microbiota transplant. One mouse from the antibiotic-treated group died for unknown reasons during the study. Mice were fed a standard diet until 1 week before fecal transplantation that was substituted by purified-defined germ-free diet (AIN-93 G, Envigo).

For the fecal transplant (control, 3FN, 6FN, LNB and GNB groups) a pool mix was prepared everyday with four fecal samples, one of each infant, and was kept on ice during the process. Each mouse received a volume of 100 μl of the pool mix in 3 consecutive days through oral gavage. Regarding oligosaccharide supplementation, 100 μl of 3FN, 6FN, LNB or GNB at 10 mM were supplied through oral gavage every day to each mouse (5 mice in each feeding group) for 3 weeks. Control mice received water. Feces were collected from each mouse before the sacrifice. After this, intestines content and tissues were also collected. Feces and intestines content were kept frozen at −80°C until analysis. Intestine tissues were preserved in RNA Later (Sigma) and kept the first 24 h at 4°C and then at −80°C until use.

All animal experimentation procedures were validated by the Ethical Committee for Use of Laboratory Animals of the University of Valencia, and the Department of Agriculture, Livestock and Fisheries of the Generalitat Valenciana, with registration number 2018/VSC/PEA/0181. The use of human samples was approved by the Ethical Committee for Human Research of the University of Valencia, with registration number H1544010468380. Written informed consent was obtained from a parent of each of the subjects.

### DNA extraction from fecal samples

Total DNA was extracted from fecal samples of each mouse at the end of the experiment (Figure 1) using the MasterPure Complete DNA & RNA Purification Kit (Epicenter) according to the manufacturer’s instructions with some modifications that included a 60 min incubation with 2 μl of lysozyme 20 mg/ml and 1 μl of mutanolysin 10 U/ml followed by mechanical disruption using 3-µm diameter glass beads in a FastPrep 24-5 G Homogenizer (MP Biomedicals, CA, USA). Total DNA concentration was measured using a Qubit® 3.0 Fluorometer (Life Technologies, Carlsbad, CA, United States) and DNA integrity-quality was analyzed by gel electrophoresis. A DNA sample from the control group was discarded for failing to pass the quality control.

### 16S rRNA amplicon sequencing and data analysis

The amplification of the V3-V4 variable region of the 16S rRNA gene of total DNA from fecal samples was conducted following the Metagenomic Sequencing Library Preparation Illumina protocol (Cod. 15044223 Rev. A). Gene-specific primers (PCR1_f: 5′-TCGTCGGCAGCGTCAGATGTGTATAAGAGACAGCCTACGGGNGGCWGCAG-3′; PCR1_r: 5′-GTCTCGTGGGCTCGGAGATGTGTATAAGAGACAGGACTACHVGGGTA

TCTAATCC-3′) containing Illumina adapter overhang nucleotide sequences were selected as previously described.^78^ A multiplexing step was performed using Nextera XT Index Kit and a Bioanalyzer DNA 1000 chip (Agilent Technologies) was used to verify the amplicons size (~550 bp). Libraries were sequenced using a 2 × 300 pb paired-end run (MiSeq Reagent kit v3) on a MiSeq Sequencer according to manufacturer’s instructions (Illumina) by the Central Service of Research Support of the University of Valencia (Spain).

Sequencing data have been demultiplexed using Illumina bcl2fastq© program. Forward and reverse raw reads were checked for quality, adapter trimmed and filtered using AfterQC^79^ and FastQC v0.11.8 (http://www.bioinformatics.babraham.ac.uk) tools. QIIME software V1.9.1 was used to analyze the MiSeq sequencing data,^80^ including forward and reverse reads joining, chimera removal, data filtering and taxonomic annotation. Chimeric sequences were removed from the reads using the USEARCH 6.1 algorithm.^81^ Reads were clustered into operational taxonomic units (OTUs) based on a 97% identity threshold value. Alignment of the sequences was carried out using PyNAST^82^ with reference to the Greengenes core reference database (version 13_8).^83^ Taxonomic assignment was made using the UCLUST classifier.^81^

Microbiota data were analyzed in the Calypso online platform (v8.84) (http://cgenome.net/wiki/index.php/Calypso/) and data was normalized by the Total-Sum Scaling (TSS) method with square root transformation. Total Richness and alpha diversity indexes (Shannon and Chao1) were determined. Beta diversity was represented by PCoA plot based on Bray–Curtis and Jaccard distance. Analysis of similarities (ANOSIM) and permutational multivariate analysis of variance (Adonis) based on Bray–Curtis distance were also achieved. Linear discriminant analysis effect size (LEfSe) was used to identify differences in microbial genera and families between control and oligosaccharide treated mice groups.

Raw sequences are deposited into the Sequence Read Archive (SRA) of NCIB (http://www.ncbi.nlm.nih.gov/sra) and can be assessed with the accession number PRJNA668130.

## Quantification of *Bifidobacterium* genus by specific real-time PCR

Quantitative real-time PCR (qPCR) assays were performed as previously described^24^ and using the *Bifidobacterium*-specific 16S rRNA gene primers Bifido5ʹ (GAT TCT GGC TCA GGA TGA) and Bifido3ʹ (CTG ATA GGA CGC GAC CCC). The qPCR amplification and detection were conducted in a LightCycler 480 Real-Time PCR System (Roche). Each reaction mixture of 10 μl contained NZYSpeedy qPCR Green Master Mix (NZytech), 0.25 μl of each primer (10 μM) and 1 μl of template DNA. All samples were analyzed in triplicate. Standard curves of specific DNA amplicon-fragments obtained with the primers pair were used to calculate bacterial concentration in each sample.

### Short-chain fatty acids’ (SCFAs) analysis

SCFAs were extracted from large intestine content and analyzed by gas chromatography mass spectrometry (GC/MS) as described in the Agilent application note.^84^ Briefly, 30 mg of intestinal content were suspended in 1 ml 10% isobutanol and mechanically homogenized with glass beads The mixtures were centrifuged at 17,000 *g* for 5 min. Sample supernatants and standards were treated and subjected to the derivatization procedure as described in the Agilent application note, and 3-methylpentanoic acid was used as internal standard. Analysis of SCFAs was performed on an Agilent 7890B GC/5977 MSD (Agilent, Santa Clara, CA, USA) using a Agilent HP-5 ms column (30 m × 0.25 mm × 0.25 µm). The injector temperature was set at 260°C in split mode (10:1) and a volume of 1 µl was automatically set. The column temperature was initially 40°C for 5 min and then increased to 120°C at 10°C/min and then ramped to 310°C at 40°C/min and held for 2 min. The MS transfer line was maintained at 280°C and the ion source at 230°C.

### Serum lipid analysis

About 1 ml of blood from the heart of mice was collected at the time of sacrifice and then centrifuged at 1,500 × *g* for 5 min. The serum was used to determine total cholesterol, triglycerides and low-density lipoproteins (LDL) by Echevarne Laboratories (Spain) using standard methods.

### RNA isolation

Five mg of large intestine tissue were homogenized using a Polytron PT10-35 GT (Thermo Fisher Scientific). After tissue disruption, the RNA was purified using the NZY total RNA isolation kit (Nzytech) following the manufacturer instructions. RNA quality was checked by gel electrophoresis and quantified spectroscopically using a NanoDrop ND-1000 system (NanoDrop Technologies).

### Analysis of cytokines and Toll-like receptors expression

The expression of genes encoding cytokines (IL-1β, IL-6, Cxcl15 (IL-8), IL-12, TNF-α, IFN-γ, IL-4, IL-10 y IL-13) and Toll-like receptors (TLR2 y TLR4) were evaluated using reverse transcription-quantitative PCR (RT-qPCR). First-strand complementary DNA (cDNA) was obtained from 1 μg of total RNA using Maxima first-strand cDNA synthesis kit (Thermo Scientific™). RT-qPCR was performed for each cDNA in triplicate using the LightCycler 480 System (Roche Technologies). Each qPCR reaction mixture (10 μl) contained 5 μl of NZY Speedy qPCR Green Master Mix 2X (Nzytech), 0.4 μl of each primer (10 mM) and 2 μl of diluted 1:20 cDNA template. Primer sequences are listed in Supplemental Table 3. The expression level of GAPDH and RPLPO housekeeping genes was used as reference.

The qPCR conditions were 95°C for 2 min, followed by 40 cycles of 10 s denaturation at 95°C and 15 s of annealing/extension at 60°C or 65°C (Supplemental Table 3). Relative expression values were calculated using the Relative Expression Software Tool (REST 2009, Qiagen). Linearity and amplification efficiency were determined for each primer pair using LinRegPCR software.^85^

### Culture of *Bifidobacterium adolescentis* with LNB and GNB

The growth of *B. adolescentis* ATCC 15703 in the presence of each disaccharide at 10 mM was tested in MRS basal medium as previously described.^23^

### Statistical analysis

The data obtained were analyzed by one-way ANOVA with Dunnett´s multiple comparisons test using GraphPad Prism, version 6.07 (GraphPad Software Inc., San Diego, CA, USA). Correlation of the expression levels of cytokines with butyrate concentrations were analyzed using the Spearman’s correlation coefficient. Statistical significant differences were accepted at different levels # *p* < 0.1, *p < 0.05; **P < 0.01; ***p < 0.001, ****p < 0.0001.

## Supplementary Material

Supplemental Material
