# Supplementary material for: Infant gut microbiota modulation by human milk disaccharides in humanized microbiome mice
Source: Gut Microbes. 2021 May 3;13(1):1914377. doi: 10.1080/19490976.2021.1914377 (PMC8096338; doi:10.1080/19490976.2021.1914377)
Supplement: Supplemental Material [file KGMI_A_1914377_SM8505.zip › Supplementary information/Supplemental_Figure 1.pdf]

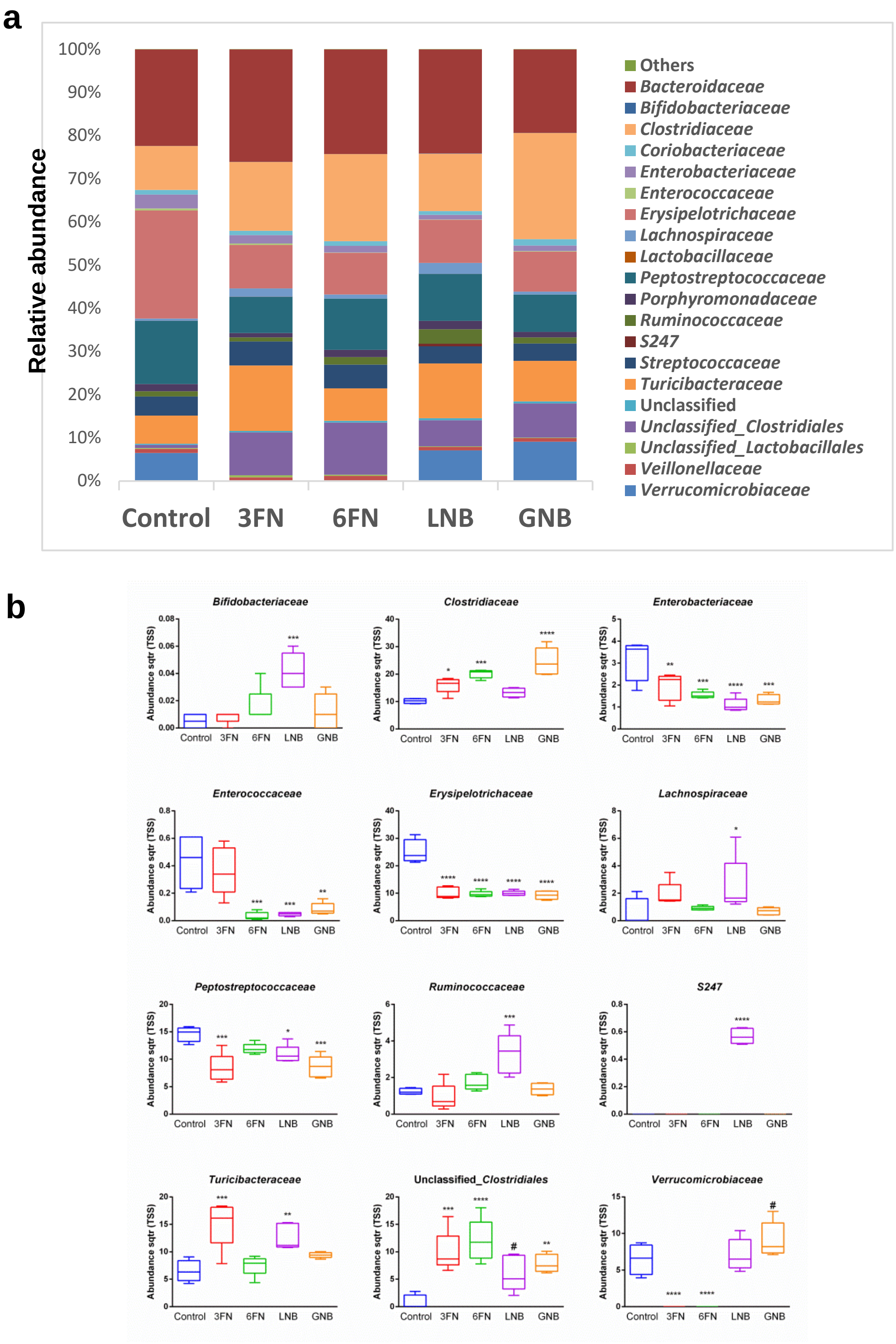

Supplemental Figure 1. Effect of fucosyl-α-1,3-*N*-acetylglucosamine (3FN), fucosyl-α-1,6-*N*-acetylglucosamine (6FN), lacto-*N*-biose (LNB) or galacto-*N*-biose (GNB) on mice gut microbiota at family level. (a) Microbial relative abundance. Bars represent each diet group and values are mean relative abundance of each bacterial genus. (b) Box plot of relative abundances of the statistically significant different families after oligosaccharide treatments. Box plots present the median (interquartile range) and min/max., n=4 (control group); n=5 (diet group). Statistically significant differences compared to control are indicated: # p<0.1, *p < 0.05; **P<0.01; ***p<0.001, ****p<0.0001
