# Supplementary material for: Infant gut microbiota modulation by human milk disaccharides in humanized microbiome mice
Source: Gut Microbes. 2021 May 3;13(1):1914377. doi: 10.1080/19490976.2021.1914377 (PMC8096338; doi:10.1080/19490976.2021.1914377)
Supplement: Supplemental Material [file KGMI_A_1914377_SM8505.zip › Supplementary information/Supplemental_Figure 2.pdf]

a

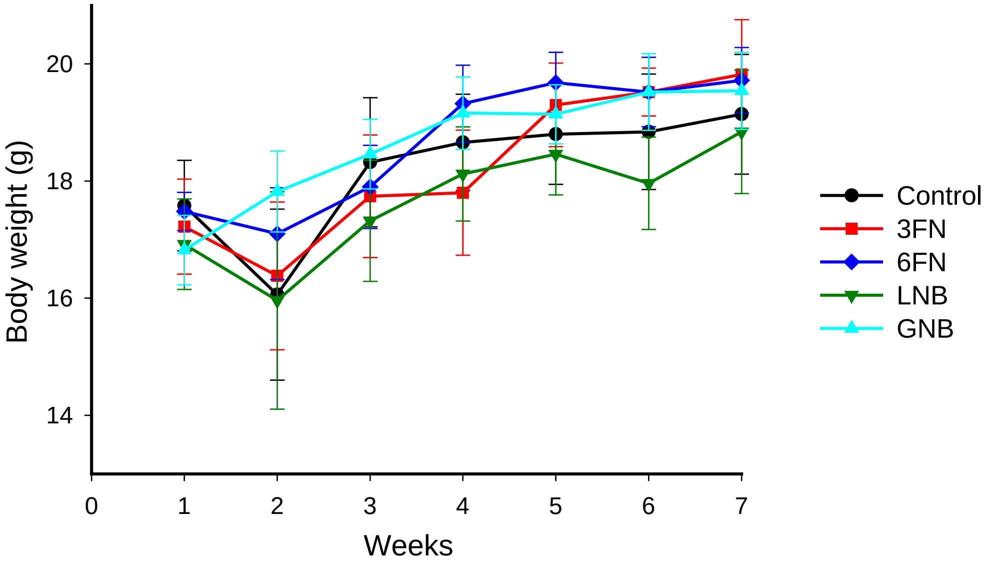


b

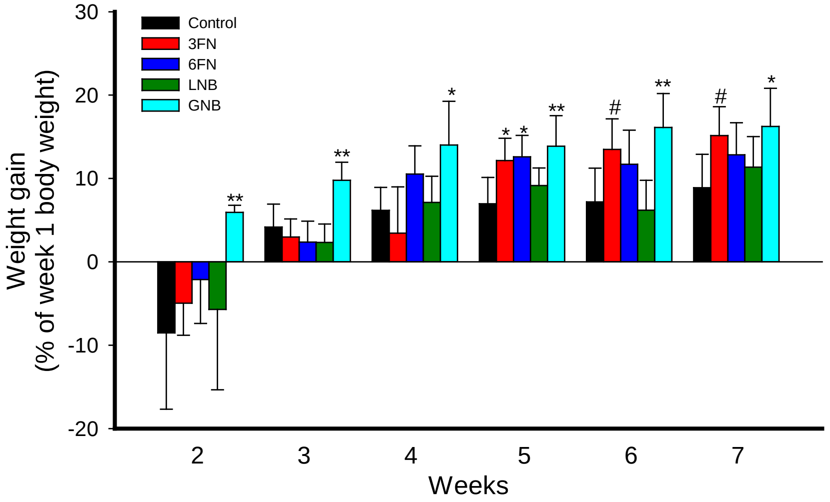


**Supplemental Figure 2**. Effect of fucosyl-α-1,3-*N*-acetylglucosamine (3FN), fucosyl-α-1,6-*N*-acetylglucosamine (6FN), lacto-*N*-biose (LNB) or galacto-*N*-biose (GNB) on mice body weigh. (a) Body weight. Data presented are mean values and error bars indicate standard deviations, n=5 (control group); n=5 (diet group). No significant statistical differences were detected between groups by one-way ANOVA with Dunnett´s correction. (b) Body weight gain as a percentage of week 1 body weight. Data presented are mean values and error bars indicate standard deviations, n=5 (control group); n=5 (diet group). Statistically significant differences compared to control were detected by one-way ANOVA with Dunnett´s correction and they are indicated: # p<0.1, *$p$ < 0.05; **P<0.01; ***p<0.001, ****p<0.0001.
