# Supplementary material for: Infant gut microbiota modulation by human milk disaccharides in humanized microbiome mice
Source: Gut Microbes. 2021 May 3;13(1):1914377. doi: 10.1080/19490976.2021.1914377 (PMC8096338; doi:10.1080/19490976.2021.1914377)
Supplement: Supplemental Material [file KGMI_A_1914377_SM8505.zip › Supplementary information/Supplemental_Figure 3.pdf]

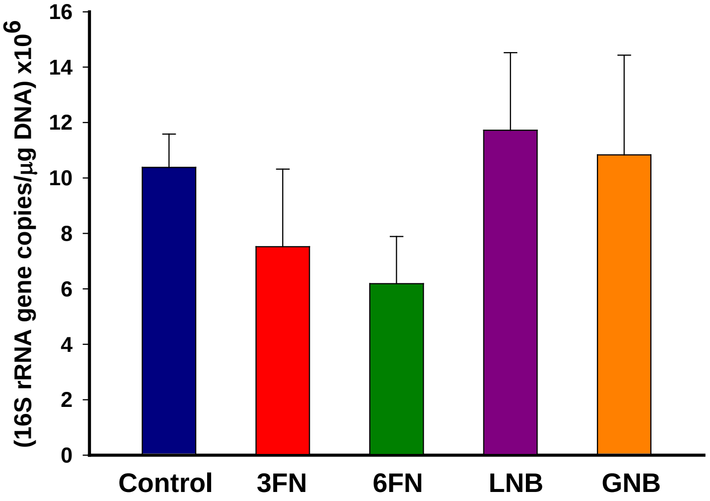

Supplemental Figure 3. *Bifidobacterium* genus levels measured with qPCR in mice fecal microbiota. Diet groups: control, fucosyl-α-1,3-*N*-acetylglucosamine (3FN), fucosyl-α-1,6-*N*-acetylglucosamine (6FN), lacto-*N*-biose (LNB) and galacto-*N*-biose (GNB). Data presented are mean values and error bars indicate standard deviations, n=4 (control group); n=5 (diet group). No significant statistical differences were detected between the control and each diet group by one-way ANOVA with Dunnett´s correction.
