# Supplementary material for: Infant gut microbiota modulation by human milk disaccharides in humanized microbiome mice
Source: Gut Microbes. 2021 May 3;13(1):1914377. doi: 10.1080/19490976.2021.1914377 (PMC8096338; doi:10.1080/19490976.2021.1914377)
Supplement: Supplemental Material [file KGMI_A_1914377_SM8505.zip › Supplementary information/Supplemental_Table 3.pdf]

Supplemental Table 3. Primers used in this study

Target	Primer name	Sequence	Annealing/ extension Temperature	Reference
IL-1β	IL-1β-F	5’-TCGCTCAGGGTCACAAGAAA-3’	60ºC	(1)
	IL-1β-R	5’-CATCAGAGGCAAGGAGGAAAAC-3’		
IL-4	IL-4-F	5’-GGTCTCAACCCCCAGCTAGT-3’	65ºC	(2)
	IL-4-R	5’-GCCGATGATCTCTCTCAAGTGAT-3’		
IL-6	IL-6-F	5’-CTGCAAGAGACTTCCATCCAGTT-3’	65ºC	(1)
	IL-6-R	5’-GAAGTAGGGAAGGCCGTGG-3’		
Cxcl15 (IL-8)	IL-8-F	5’-CAGGCCACAGACGGACATG-3’	60ºC	(1)
	IL-8-R	5’-GGACGAAGATGCCTAGGTTAAGG-3’		
IL-10	IL-10-F	5’-GCTCTTACTGACTGGCATGAG-3’	65ºC	(1)
	IL-10-R	5’-CGCAGCTCTAGGAGCATGTG-3’		
IL-12 (IL-12p40)	IL-12-F	5’-AGCACCAGCTTCTTCATCAGG-3’	60ºC	(3)
	IL-12-R	5’-CCTTTCTGGTTACACCCCTCC-3’		
IL-13	IL-13-F	5’-CCTGGCTCTTGCTTGCCTT-3’	65ºC	(2)
	IL-13-R	5’-GGTCTTGTGTGATGTTGCTCA-3’		
TNF-α	TNF-α –F	5’-CCACCACGCTCTTCTGTCTAC-3’	65ºC	(1)
	TNF-α-R	5’-TGGGCTACAGGCTTGTCACT-3’		
INF-γ	INF-γ-F	5’-CATTCATGAGTATTGCCAAGTTTG-3’	60ºC	(4)
	INF-γ-R	5’-GCTGGATTCCGGCAACAG-3’		
TLR2	TLR2-F	5’- GTCTCTGCGACCTAGAAGTGGA -3’	65ºC	(5)
	TLR2-R	5’-CGGAGGGAATAGAGGTGAAAG A-3’		
TLR4	TLR4-F	5’-AGCAGAGGAGAAAGCATCTATGATGC-3’	65ºC	(5)
	TLR4-R	5’-GGTTTAGGCCCCAGAGTTTTTCTCC-3’		
GAPDH	GAPDH-F	5’-AGCTTGTCATCAACGGGAAG-3’	65ºC	(1)
	GAPDH-R	5’-TTTGATGTTAGTGGGGTCTCG-3’		
RPLP0	RPLP0-F	5’-AGATTCGGGATATGCTGTTGGC-3’	65ºC	(6)
	RPLP0-R	5’-TCGGGTCCTAGACCAGTGTTC-3’		

References:

1. Leclercq S, Mian FM, Stanisz AM, et al. Low-dose penicillin in early life induces long-term changes in murine gut microbiota, brain cytokines and behavior. Nat Commun. **2017**; 8(1):15062.

2. Spandidos A, Wang X, Wang H, Seed B. PrimerBank: a resource of human and mouse PCR primer pairs for gene expression detection and quantification. Nucleic Acids Res. **2010**; 38:D792-9.

3. Cardona P-J, Gordillo S, Diaz J, et al. Widespread Bronchogenic Dissemination Makes DBA/2 Mice More Susceptible than C57BL/6 Mice to Experimental Aerosol Infection with Mycobacterium tuberculosis. Infect Immun. **2003**; 71(10):5845–5854.

4. Keilbaugh SA, Shin ME, Banchereau RF, et al. Activation of RegIIIbeta/gamma and interferon gamma expression in the intestinal tract of SCID mice: an innate response to bacterial colonisation of the gut. Gut. **2005**; 54(5):623–9.

5. An H, Yu Y, Zhang M, et al. Involvement of ERK, p38 and NF-kappaB signal transduction in regulation of TLR2, TLR4 and TLR9 gene expression induced by lipopolysaccharide in mouse dendritic cells. Immunology. **2002**; 106(1):38–45.

6. Xu L, Ma X, Cui B, Li X, Ning G, Wang S. Selection of reference genes for qRT-PCR in high fat diet-induced hepatic steatosis mice model. Mol Biotechnol. **2011**; 48(3):255–262.
